# Application of text mining to develop AOP-based mucus hypersecretion genesets and confirmation with in vitro and clinical samples

**DOI:** 10.1038/s41598-021-85345-9

**Published:** 2021-03-17

**Authors:** Emmanuel Minet, Linsey E. Haswell, Sarah Corke, Anisha Banerjee, Andrew Baxter, Ivan Verrastro, Francisco De Abreu e Lima, Tomasz Jaunky, Simone Santopietro, Damien Breheny, Marianna D. Gaça

**Affiliations:** British American Tobacco, GR&D Centre, Regents Park Road, Southampton, SO15 8TL UK

**Keywords:** Systems biology, Gene expression analysis

## Abstract

Mucus hypersecretion contributes to lung function impairment observed in COPD (chronic obstructive pulmonary disease), a tobacco smoking-related disease. A detailed mucus hypersecretion adverse outcome pathway (AOP) has been constructed from literature reviews, experimental and clinical data, mapping key events (KEs) across biological organisational hierarchy leading to an adverse outcome. AOPs can guide the development of biomarkers that are potentially predictive of diseases and support the assessment frameworks of nicotine products including electronic cigarettes. Here, we describe a method employing manual literature curation supported by a focused automated text mining approach to identify genes involved in 5 KEs contributing to decreased lung function observed in tobacco-related COPD. KE genesets were subsequently confirmed by unsupervised clustering against 3 different transcriptomic datasets including (1) in vitro acute cigarette smoke and e-cigarette aerosol exposure, (2) in vitro repeated incubation with IL-13, and (3) lung biopsies from COPD and healthy patients. The 5 KE genesets were demonstrated to be predictive of cigarette smoke exposure and mucus hypersecretion in vitro, and less conclusively predict the COPD status of lung biopsies. In conclusion, using a focused automated text mining and curation approach with experimental and clinical data supports the development of risk assessment strategies utilising AOPs.

## Introduction

Cigarette smoking is a leading cause of mortality and a major contributor to cardiovascular diseases, chronic obstructive pulmonary disease (COPD), and lung cancer. The introduction of new nicotine delivery devices, including electronic cigarette and tobacco heated products offers smokers potentially reduced risk alternatives compared to combustible tobacco products. The longer-term risk associated with these potentially reduced risk alternatives is not yet fully understood, and their rapid diversification is adding further demands in terms of risk characterization and stewardship of ingredients. In 2017, Murphy et al.^1^ proposed an assessment framework for modified risk tobacco and nicotine products focussed on chemical emissions, pre-clinical, clinical, and population studies. The pre-clinical studies consisting of:

1. in vitro regulatory toxicology including genotoxicity testing such as Ames, mouse lymphoma assays, neutral red cytotoxicity testing^2^.
2. in vitro modelling of tobacco related-disease endpoints including cancer (transformation assays), cardiovascular (monocyte adhesion, endothelial wound healing), and COPD (goblet cell hyperplasia)^3–6^.

Regulatory toxicology assays and in vitro models of disease rely on apical endpoints and do not provide mechanistic information on the mode of action of the different products’ aerosols. Thus, the proposed assessment framework by Murphy et al.^1^ incorporates systems biology. Top down omics approaches are unbiased since there is no single end-point pre-selected. Furthermore, they are well suited to provide mechanistic information since they can be applied to a variety of sub-cellular matrices such as RNA, proteins, and metabolites.

Data generated from systems biology approaches can yield an abundance of multi-layered information beyond expression levels. Knowledge-base repositories including KEGG^7–9^, GO, and the Reactome^10^ catalogue genes, pathways and their corresponding biological function. These repositories can be mined to match experimental gene expression profiles with biological functions to derive mechanistic understandings of the effects caused by a treatment. Current analysis tools for systems biology are, however, poorly tailored towards tobacco product toxicity and diseases. Adverse outcome pathways (AOPs) frameworks however, can combine datasets from systems biology discovery along a series of key events (KE) to confirm or define new genesets with better relevancy to diseases, including tobacco-related diseases^11,12^.

An AOP framework describes the causal link between a molecular initiating event (MIE) followed by a series of KEs organized across a biological hierarchy from the molecular to the cellular, organ, individual, and population level ultimately leading to an adverse outcome^13^. AOPs are modular constructs based on the best evidence available at the time and for which the robustness of each KE is evaluated using Bradford-Hill criteria of plausibility, essentiality, and empirical support. KEs can form the foundation of new genesets that are not driven by functional gene ontology, but that are more directly related to disease process^14^. In the field of toxicology, an AOP can help to define an “integrated testing strategy (ITS)” which could have a predictive value on the impact of chemicals on human health and or on the environment (eco-toxicology)^13^.

In 2017, Luettich et al*.*^12^*,* described an AOP for mucus hypersecretion (AOP ID 148). This phenotype is symptomatic of COPD, asthma, and, for some elements, cystic fibrosis. COPD is a tobacco smoking-related condition and is predicted to become a leading cause of mortality in the twenty-first century due to the added contribution of air pollution^15^. COPD is a complex, progressive, and non-reversible disease characterized by airflow limitation and 5 stages of severity. The reduction of the respiratory capacity is due, amongst other, to airway tissue remodelling and mucus hypersecretion.

**Figure 1:**
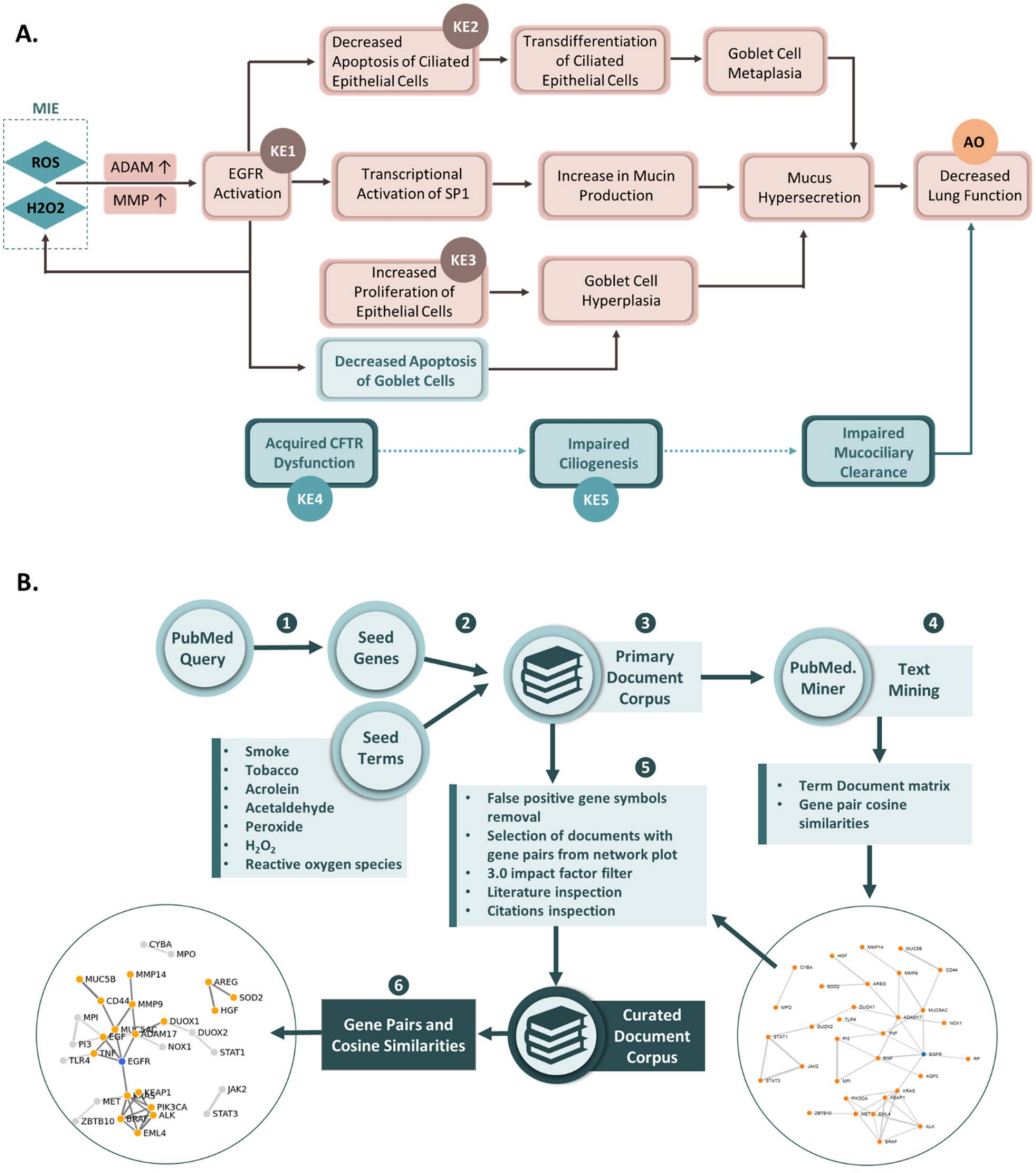
(**A**) Mucus hypersecretion AOP and impaired mucocilliary clearance key events (AOP 148). (**B**) Text mining process. 1. A manual search was first conducted using KE terms to derive seed genes. 2. Pubmed was searched using a combination of seed genes and tobacco related key terms. 3. The resulting primary document corpus was then mined for gene symbols (4) 4. Gene pairs were used to construct network plots. 5. Gene pairs from articles with an impact factor equal or higher than 3.0 were retained together with the corresponding publication. Further manual searches were conducted to include the most recent literature. 6. Gene network plots and KE genesets were finalized based on the curated document corpus.

The mucus hypersecretion AOP has 10 modules in total, one MIE, eight KEs, with a decrease in lung function as the adverse outcome^12^. There is strong evidence that free radicals and oxidative stress are the molecular initiating events leading to EGFR activation, the first key event in the mucus hypersecretion AOP. In turn, EGFR activation leads to a series of key events involved in the loss of ciliated cells, increase in the goblet cell population and ultimately mucus hypersecretion (Fig. 1A). The confidence in the relationship between the different KEs of this pathway ranges from strong, to moderate and weak^12^.

In this report, we describe the development of four bespoke genesets constructed using in silico literature mining with search terms based on 3 KEs and 2 putative KEs that contributes to the mucus hypersecretion AOP and to mucocilliary clearance impairment, respectively. A text-mining approach combined with manual curation was used to identify genes involved in each of the KEs, and to formulate interaction networks. Unsupervised hierarchical clustering was performed to assess the discrimination power of the KE genesets on two in vitro transcriptomic datasets obtained from studies in which (1) 3D reconstituted human lung tissue (MucilAir, Epithelix Sarl), were exposed to aerosols from a reference cigarette (1R6F), an electronic cigarette (IS1(TT)) or air, (2) in vitro human tracheal epithelial cells IL-13, an inducer of the mucus hypersecretion phenotype. A similar analysis was conducted on a public transcriptomic data set generated from lung tissue samples of COPD patients and healthy smokers to determine whether the genesets allowed to group samples based on the health status of the subjects. We report that the genesets suitably discriminated between in vitro cigarette smoke exposure, vapour/air exposure, and IL-13 treatments. The genesets, however, did not discriminate between health status when applied to RNA levels from lung biopsies taken from healthy and COPD subjects.

## Results

### Stage 1: Mucus hypersecretion key events literature gathering

Three key events from the mucus hypersecretion AOP (Fig. 1, adapted from Luettich et al.)^12^ and two putative key events (unpublished) involved in impaired mucus clearance were selected for the purpose of gene interaction networks building, namely:

Key Event 1 (KE1): 'EGFR Activation'.

Key Event 2 (KE2): 'Decreased apoptosis (epithelial cells)'.

Key Event 3 (KE3): 'Increased proliferation (epithelial cells)'.

Putative Key Event 4 (pKE4): 'Acquired CFTR dysfunction'.

Putative Key Event 5 (pKE5): 'Impaired ciliogenesis'.

**Figure 2:**
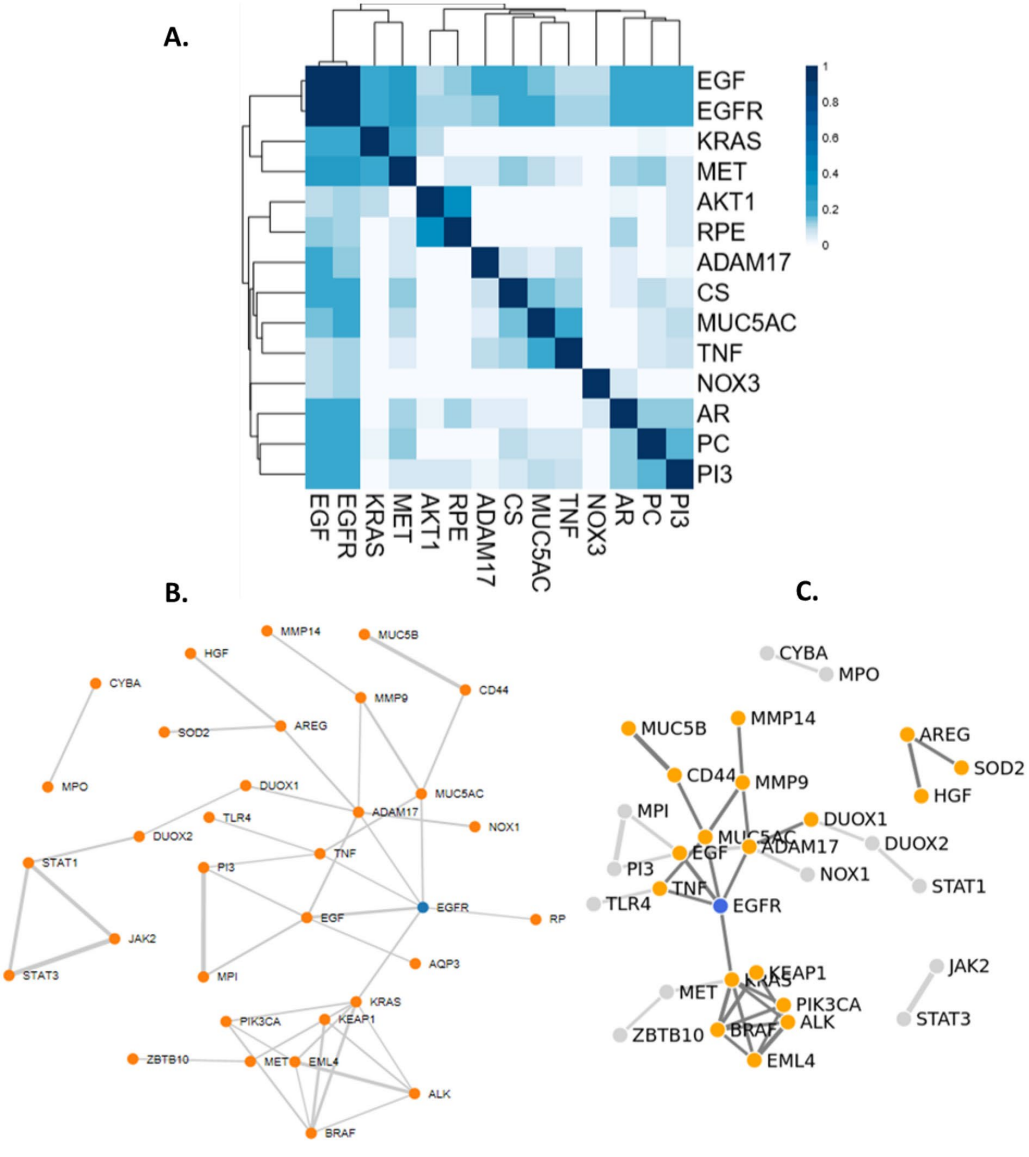
(**A**) Cosine-similarity matrix for KE1 seed genes. (**B**) Network plot established from the document corpus using a cosine similarity threshold > 0.1. (**C**) Network plot established using the curated document corpus and a cosine similarity threshold > 0.1.

The seed gene (S-genes) list for each of these key events is shown in Supplementary Table S1 and used in combination with the key terms “smoke” OR “tobacco” OR “acrolein” OR “acetaldehyde” OR “peroxide” OR “H2O2” OR “reactive oxygen species” to mine relevant abstracts in NCBI PubMed. The resulting abstracts accessible in the evidence tables (Supplementary Tables S2–S6) formed the document corpus and were processed using the R package pubmed.mineR^16^ to find co-occurrences of each gene on this list with every other gene on the same list, with each gene pair mentioned being ranked by cosine similarity^17^. Cosine similarity of gene co-occurrences was used as a score to assess potential gene interactions with 0 meaning no co-occurrence and 1 meaning 100% co-occurrence in the document corpus. Table 1 summarizes some of the key metrics from the primary document corpus. An example of the KE1 resulting cosine similarity interaction matrix for the seed genes is shown in Fig. 2A alongside the corresponding interaction network (Fig. 2B). Each KE integration matrix for the top 60 gene pairs is presented in Supplementary Figure S6 and the cosine similarity for each gene pair is given in Supplementary Tables S7–S11.

**Table 1:** Summary of literature reference, gene pairs pre- (Stage 1) and post- (Stage 2) curation used for building the key event (KE) networks.

	Primary document corpus			Curated corpus			
	Number of seed genes	Number of abstract retrieved	Total number of unique genes from retrieved abstracts	Number of abstracts after curation	Total number of unique genes after curation	Unique genes in detail	List of gene pairs
KE1	1	718	215	165 + 12^a^	25	S-genes: 1; R-genes: 18; A-genes: 6	See Table S12
KE2	21	17,570	1242	244 + 6^a^	KE2: 35; KE2-KE3: 124	S-genes: 20 (one duplicate removed); R-genes: 8; A-genes: 7	See Table S13
KE3	84	42,465	1873	265 + 6^a^	KE3: 106; KE2-KE3: 124	S-genes: 84; R-genes: 16; A-genes: 6	See Table S14
KE4	34	2115	516	80 + 11^a^	50	S-genes: 34; R-genes: 13; A-genes: 3	See Table S15
KE5	44	192	114	7 + 13^a^	51	S-genes: 42 (one duplicate and one false acronym removed); R-genes: 0; A-genes: 9	See Table S16

^a^Indicates number of genes that were added after a further manual inspection of key references.

### Stage 2: Mucus hypersecretion key event literature curation

For each KE, the abstracts describing a gene pair and published in journals with impact factors above 3.0 were retained and inspected to confirm the associated gene pairs and remove false positive, e.g. due to the use of acronyms redundant of gene names. Manual literature review focused primarily on publications from journals with an impact factor > 5.0. Abstracts, introductions, discussions were studied in more detail until the relevance of the paper in terms of the AOP could be established. Next, if a given gene pair of interest was not present in any of the highest-ranking abstracts, the publications from journals with an impact factor between 3.0 and 5.0 were also inspected. The manual review of these papers focused mainly on abstracts. Once it was established that the paper included relevant information on the gene pairs that otherwise could not be annotated, the rest of the paper (introduction, results, discussion) was also analysed. Only references with genes using HGNC-approved symbols were further carried to this stage and formed the curated document corpus.

The gene pair (co-occurrence) number confirmed for each KE is summarized in Table 1 and the associated genes were grouped in a category labelled “R-genes” (Relevant-genes). The detail of the literature reference and associated gene pairs is given in Supplementary Tables S12–S16. Each gene pair of interest has been annotated with biological pathways and processes, molecular interactions, cell- and tissue-specific expression or protein subcellular location information available through public resource databases (KEGG, IntAct, Reactome). Biological information shared among both members of a pair is reported in a table of top-scoring co-occurring gene pairs presented in Supplementary Tables S12–S16. A further manual search was conducted from review article citations to identify the most relevant references to complete the curated document corpus. A few more genes were retrieved from this last step and were put in the category “A-genes” (Additional genes) and the gene list and references are accessible in Supplementary Table S17. For each KE, genes from the co-occurring pairs (R-genes) were pooled with the seed genes (S-genes) and the “A-genes” to obtain KE-specific genesets. The gene list for KE2 and KE3 were merged due to the important overlap found between these two key events. The full list of KE genes is given in Supplementary Tables S18–S21. Only four KE genesets are described since KE2 and KE3 genes were pooled due to the degree of redundancies in contributing genes.

KE1 is used as an illustration. In KE1, one seed gene was pre-defined (S-gene), 22 gene pairs were found post-curation of the document corpus connecting 18 genes (R-genes) (Fig. 2C—blue genes connections). Another 6 genes (A-genes) were included from the additional literature review post-curation from a collection of 12 references retrieved manually. The resulting total of S-gene (1) + relevant genes in pairs (18) + additional genes from ad hoc searches (6) gives 25 genes included in the KE1 geneset.

### Stage 3: Key event genesets validation

**Figure 3:**
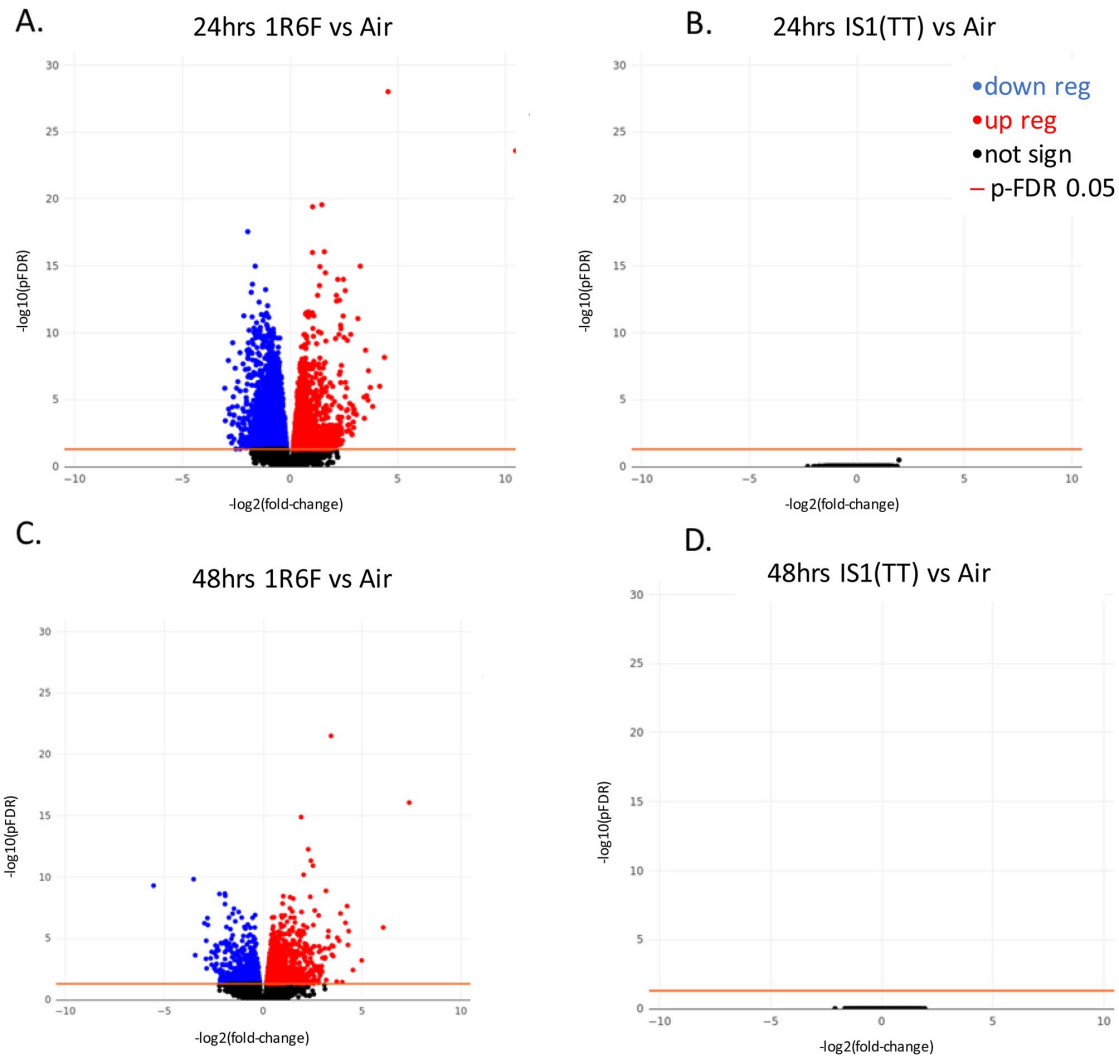
Volcano plots for the following 4 RNA-seq contrasts with pFDR < 0.05 threshold. 1R6F cigarette smoke exposure (1/30 dilution) vs air control, 24 hrs (**A**) and 48 hrs (**C**) post-exposure recovery, respectively. IS1 (TT) electronic cigarette aerosol exposure (1/3 dilution) vs air control, 24 hrs (**B**) and 48 hrs (**D**) post-exposure recovery, respectively.

**Figure 4:**
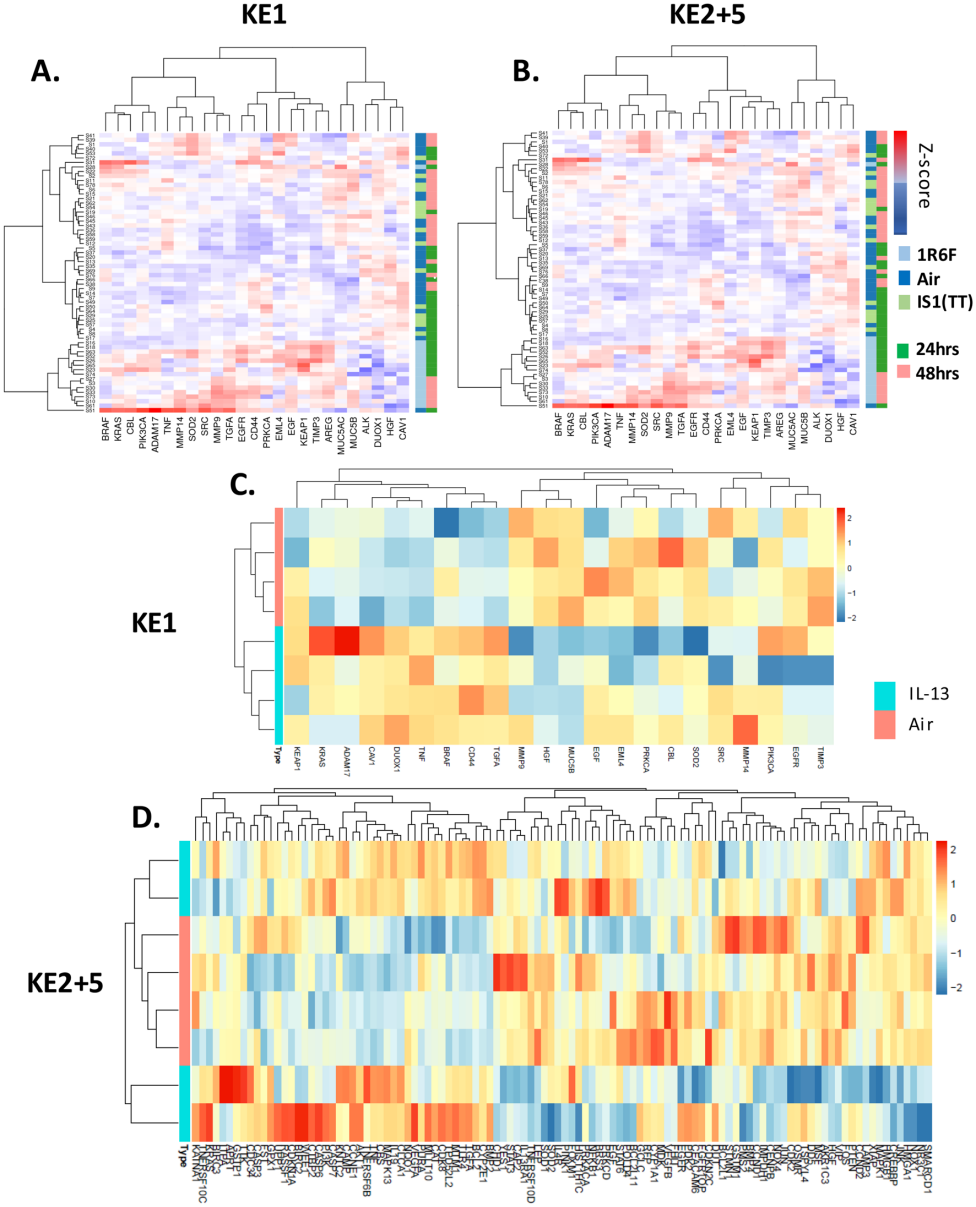
Unsupervised clustering using two in vitro datasets and the KE genesets. (**A**) and (**B**) show clustering using the acute exposure (1 hrs) to cigarette smoke, e-cigarette aerosol, and air with 24 and 48 hrs post-exposure recovery for KE1 and KE2 + 5 genesets, respectively. (**C**) and (**D**) show unsupervised clustering using the 21-day incubation with IL-13 and corresponding air control with KE1 and KE2 + 5 genesets, respectively.

**Figure 5:**
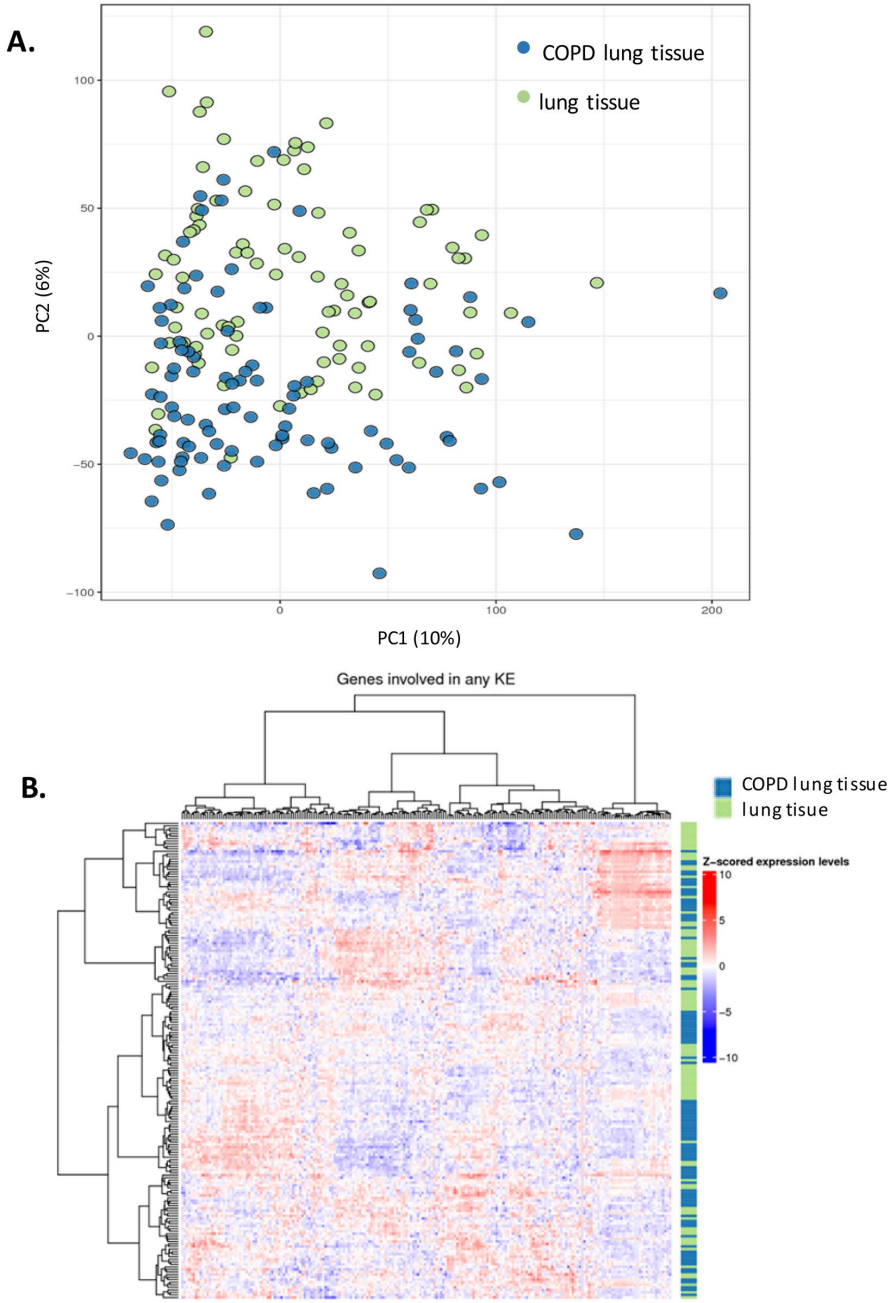
Application of the KE genesets to samples from COPD and non-COPD lung biopsies. (**A**) Principal component analysis comparing the whole normalized RNA-seq data from the COPD and non-COPD samples. (**B**) Unsupervised clustering using the COPD and non-COPD gene expression data and the pooled genesets from the five KEs.

**Figure 6:**
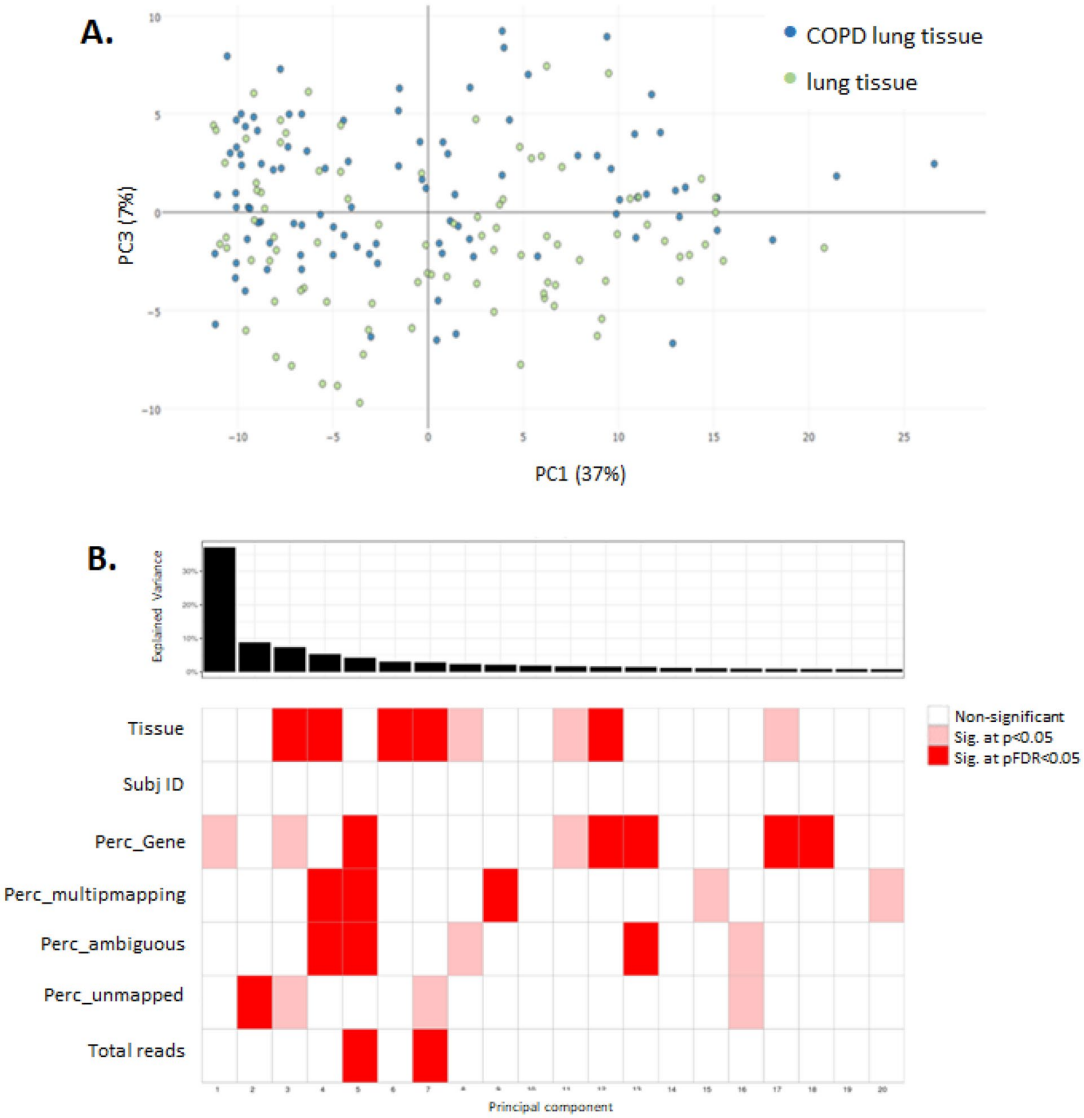
Principal component analysis with the COPD and non-COPD lung samples using the key event genes expression data (**A**) and corresponding covariate explaining the variance (**B**). *Perc_ambiguous* Percentage of read-pairs mapped to one location in the genome and associated with more than one gene. *Perc_gene* Percentage of read-pairs mapped to one location in the genome and unambiguously associated with a single gene. These are used in the differential expression analysis. *Perc_multimapping* Percentage of read-pairs mapped to more than one location in the genome. *Perc_unmapped* Percentage of read-pairs not mapped to any location in the genome.

3 datasets were used to conduct the validation step using data from (1) in vitro 3D MucilAir™ human airway cells exposed acutely to cigarette smoke and e-cigarette aerosol, (2) in vitro human tracheal epithelial cells treated for 21 days with IL-13^18^, and (3) COPD patient biopsies^19^. The validation analyses are described below for each dataset.

i. *3D human airway cells acute *in vitro* exposure to cigarette smoke and e-cigarette aerosol.* This experiment was conducted in house and the sequencing data is accessible on NCBI SRA. Cells were exposed for one hour at the air liquid interface to air, cigarette smoke or electronic cigarette vapour. The following aerosol dilution settings 1/30 1R6F, 1/3 IS(TT1) were used to achieve equivalent nicotine delivery and cell viability above 80% at 24 hrs and 48 hrs post-exposure. The rationale for selecting these dilutions is detailed in Haswell et al*.*^20^. The tissue QC data are shown in Supplementary Figs. S3 to S5, including expression of goblet, ciliated, and basal cell markers, ciliary beat frequency (CBF) and LDH release. Nicotine measured in the in vitro cell exposure chamber media is presented in Supplementary Fig. S2. RNA-sequencing was performed on samples taken at 24 hrs and 48 hrs post-exposure. Results from the differential gene expression contrasting air vs cigarette smoke, air vs e-cigarette aerosol at 24 hrs and 48 hrs post-exposure are summarized in the volcano plots shown in Fig. 3. The full list of differentially expressed genes with selected pFDR and fold change thresholds is given in Supplementary Tables S22–S25. Heatmaps showing the sample-specific expression levels of "Mucus Hypersecretion" AOP-related genes demonstrated that cigarette smoke-treated samples cluster together irrespective of the KE (Fig. 4A and Supplementary Fig. S7). Notably, the two treatment time-based subgroups of 1R6F-exposed samples (24 hrs and 48 hrs) are also found together within the sub clusters of the distance dendrogram (Fig. 4A,B and Supplementary Fig. S7). Clustering of samples according to the recovery time post-treatment (24 hrs and 48 hrs) is particularly evident, when the analysis is based on the expression levels of genes involved in decreased apoptosis or increased proliferation of epithelial cells (Supplementary Fig. S7). In total, the expression of 24 "Mucus Hypersecretion" AOP-associated genes was significantly changed by more than two-fold in response to 1R6F treatment at an adjusted p-value (pFDR) < 0.05.
ii. *Human tracheal epithelial cells exposed to IL-13 repeated treatment.* We performed an unsupervised clustering using our KE genesets and a microarray dataset from human tracheal epithelial cells grown at the air liquid interface untreated and treated with IL-13 for 21 days^18^ (GSE37693). The repeated IL-13 treatment leads to a typical mucus hypersecretion phenotype akin of the phenotype observed in inflammatory lung diseases such as asthma and COPD. Using this approach, we found that our genesets were able to discriminate the IL-13 treated samples from the untreated samples (Fig. 4C,D and Supplementary Fig. S8). For all four KE genesets (KE1, KE2-3, KE4, KE5) clustering was observed based on treatment.
iii. *Human COPD lung tissue biopsies.* This study analyses the expression levels of "Mucus Hypersecretion" AOP-associated genes in lung tissue samples obtained from 98 patients with COPD and 91 non-COPD subjects. All subjects had been diagnosed with lung cancer. RNA-seq dataset from lung biopsies collected during this study were downloaded from SRA PRJNA245811^19^. Exploratory analysis revealed that subjects cluster by tissue disease status (COPD vs normal) on the second principal component (Fig. 5A). Heatmaps of tissue-specific expression levels of "Mucus Hypersecretion" AOP-related genes showed no obvious clustering of lung tissue samples by their disease status (Fig. 5B). Principle component analysis (PCA) analyses revealed a potential disease status-effect on the expression of KE-associated genes (Fig. 6A), however, the affected principal components (second, third or fourth) were also influenced by confounding technical factors/quality of sequencing (perc gene, perc unmapped) (Fig. 6B). Perc_gene and perc_unmapped are percentage of read-pairs mapped to one location in the genome and unambiguously associated with a single gene and percentage of read-pairs not mapped to any location in the genome, respectively. Therefore, it is not possible to conclude what is driving the separation.

## Discussion

AOPs are framework constructs mapping the causal link from a MIE to an AO all the way to the individual and population level^14^. Each intermediary incident at each level of biological organization (macromolecules, cells, organs) that ultimately is contributing to the adverse outcome is supported by literature evidence. Therefore, AOPs offer the biological context to frame a targeted risk assessment and testing strategy^13^. Such an assessment strategy can be applied to potentially reduced risk tobacco and nicotine delivery products and provide information on tobacco-related disease risk. Here, we used a previously published AOP for mucus hypersecretion^12^, an adverse event observed in COPD and asthma patients, to reverse engineer KEs specific genesets by leveraging literature mining process described by Rani et al.^16^. The genesets were then validated against three transcriptomics datasets representing (1) an in vitro 3D human lung tissue (MucilAir™) acute exposure to tobacco smoke or electronic cigarette aerosol, (2) a repeated incubation in vitro with IL-13 a known inducer of the mucus hypersecretion phenotype^18^, and (3) a COPD lung biopsies dataset^19^.

The first step of our geneset building exercise was to select three known key events from the published AOP on mucus hypersecretion^12^ (KE1, KE2, KE3 shown in Fig. 1A) and two putative key events contributing to mucocilliary clearance dysfunction (unpublished) (KE4, KE5, Fig. 1A). The level of organization for the selected key events were at the macromolecular and cellular levels to account for the proximity to the endpoint considered which is gene expression. Next, a manual pubmed search was conducted using the KE name to retrieve a list of “seed genes” (S-genes) (Fig. 1B, Supplementary Table S1) which formed the basis of the automated abstract search. For KE1, EGFR was already identified as the S-gene^12^ and therefore no further search was conducted. The primary document corpus was subsequently assembled from automated text mining using the seed genes (Supplementary Table S1) in combination with the terms “smoke” OR “tobacco” OR “acrolein” OR “acetaldehyde” OR “peroxide” OR “H_2_O_2_” OR “reactive oxygen species” as input in pubmed.mineR^16^. These key terms were selected because they are known terms associated with mucus hypersecretion phenotypes in the context of COPD, asthma, and respiratory irritancy^21–23^. A total of 63,060 abstracts (primary document corpus) were recovered in total for all 5 KEs (Table 1 and Supplementary Tables S2–S6) and 3960 unique gene symbols. 175 genes were cited in pairs and ranked as the top pairs for each KE based on the highest cosine similarity score (Supplementary Tables S7–S11).

A manual inspection step was subsequently performed using 761 abstracts (curated document corpus) (Supplementary Tables S12–S16) from journals in the top impact factor range of 3.0 and above and containing at least one of 175 top gene pairs based on the highest cosine similarities. The impact factor filter was introduced to achieve a more focussed set of articles prior to manual curation. The thresholds of 3.0 was selected to take into account both the estimated reach of the journal (as measured by the impact factor) and the amount of available articles matching the search terms. After the removal of abstracts with false positive gene symbols (e.g. acronyms identical to gene names) from the curated document corpus, a supervised approach was taken to provide further manual curation of available literature by inspecting articles cited in the retained publications. From this, an additional 48 abstracts were retrieved (Table 1) (Supplementary Tables S18–S21). One of the limitations of the approach is the application of an arbitrary “3.0 minimum impact factor” filter. Impact factor do not reflect the quality of any individual research and could lead to discarding highly relevant information published in more specialized journals. The resulting reduction in the number of gene pairs from 175 to 132 is moderate in contrast to an almost 80-fold reduction in the number of abstracts used to ascertain the gene relationships. This supports the idea that the curated document corpus and filter applied have not led to a considerable loss of information.

25, 124, 50, and 51 unique genes, split between S-genes, curated document corpus genes, and literature cited in the curated document corpus, were retrieved for KE1, KE2 + KE3, KE4, and KE5, respectively (Table 1) (Supplementary Tables S18–S21). 31 of these genes overlapped with the COPD gene list published by Bosse in 2012^24^ (Supplementary Fig. S9). Amongst these genes, we find IL13 and its receptor IL13RA1 which are known the be driving the mucus hypersecretion phenotype in asthma. MMPs such as MMP9 and MMP12 were also significant and key players in tissue remodelling associated with the histologic alteration in COPD^25,26^. A more modest 9 genes were mapped with a proposed asthma geneset developed by Poole et al.^27^ which was based on transcriptomic screens performed on samples from asthma patients and gene candidates from GWAS (genome wide association studies) (Supplementary Fig. S9). It is important to remember that the AOP geneset we derived from our literature mining used “tobacco”, “smoke” and “oxidative stress” search terms, therefore, it is not anticipated to retrieve asthma specific genes. Yet, some common genes are expected in the context of goblet cell hyperplasia and mucus hypersecretion occurring in both COPD and asthma.

The KE genesets were subsequently validated by unsupervised clustering against 3 different transcriptomic datasets including (1) an acute cigarette smoke and e-cigarette exposure, (2) a repeated incubation with IL-13^18^, and (3) lung biopsies from COPD and healthy patients^19^.

Using the genesets individually for KE1, KE2 + 3, KE4, KE5 both the acute smoke exposure and repeated IL-13 treatment clustered separately from the untreated controls. In the acute exposure, the air control clustered together with the e-cigarette (IS1(TT)) aerosol exposure. The genes driving the differential clustering were, but not limited to, TLR4, MUC5AC, NQO1, TIMP3, TIMP3, MMP9, MMP13, TGFA, EGR1, and DNAI2. It was not surprising to observe the clustering of cigarette smoke treated samples given that the primary document corpus search was performed with the key term “smoke” and “tobacco”. This result, however, positively confirmed that the genesets are related to tobacco smoke exposure, a key matrix in the onset of COPD. No response was observed from the IS1(TT) aerosol exposure at a p-FDR of 0.05 and below which lead to the clustering of the IS1(TT) samples with the air samples. A similar clustering effect was observed with IL-13 and air treated cells. “IL-13” was a seed gene included in the document corpus for KE2 to 5 (Supplementary Table S1), therefore, these genesets will be enriched for IL-13 responsive genes. KE1, however, only used EGFR as S-gene, yet clustering is observed for the IL-13 treated cells and non-treated cells with KE1 genes (Fig. 4C). Interestingly, IL-13 treatment is a known inducer of the mucus hypersecretion and goblet cell hyperplasia phenotype in air liquid interface cell cultures^28^. Importantly the IL-13-CLCA1-MAPK13 inflammatory response pathway involved in mucus hypersecretion has been identified to be common to asthma sufferers and COPD patients^18,29^. CLCA1 is a chloride channel specific to mucosal tissues which increased activity is associated with the expression of the mucin protein MUC5AC and activation of MAPK13. MUC5AC, CLCA1, MAPK13, IL13 and its receptor IL13RA1^28,30^. These genes are all found in our KE2 + 3 geneset for decreased apoptosis of ciliated cells and increased proliferation of epithelial cells which precedes goblet cell hyperplasia and metaplasia. CLCA1 is also one of the 6 genes (CLCA1, IL4, IL13, MMP12, TGFB1, TLR4) found in common between our KE genesets and the genesets proposed by Bosse^24^ for COPD and Poole^27^ for asthma.

When the unsupervised clustering approach was applied to a sequencing dataset from COPD lung biopsies and non-COPD lungs^19^, our proposed geneset did not offer sufficient resolution to discriminate between the groups (Fig. 5B). Although, some level of clustering was observed as illustrated by a principal component analysis (Fig. 6A) a number of confounding factors could be identified from the dataset that may have contributed to the clustering mostly related to the quality of the sequencing such as unmapped reads, and perc_gene (percentage of read-pairs mapped to one location in the genome and unambiguously associated with a single gene) (Fig. 6B). Clustering based on disease status was observed when the principal component analysis was performed on the entire gene expression data and therefore additional genes may be driving this discrimination. Kim et al*.* ^19^*,* reported a total of 2312 differentially expressed genes when comparing the expression data from COPD lung and non-COPD lung. Of these, 29 were also present in our KE-based genesets but they were not sufficient to drive the unequivocal clustering of the samples on a disease status basis. Even with 2312 differentially expressed genes the separation of the groups was not complete. Importantly, in the Kim et al.^19^ paper the COPD samples were not stratified by gold stage and it was not specified which samples were from patients with or without emphysema. This is important since KE1, KE2 and KE3 in this paper relate more specifically to phenotypes observed in COPD patients without emphysema^19^. Furthermore, 90 of the COPD subjects were all diagnosed with lung cancer and were split in one of the following three COPD treatment groups, (1) corticosteroids, (2) muscarinic receptor antagonist, and (3) beta-agonist which will also impact gene expression profile. This illustrates the complexity of validating genesets for COPD, a progressive heterogenous disease which can be further confounded by the variety of medical approaches. Transcriptomics applied to biopsies of COPD patients who were stratified between Gold stage 1 to 5 with or without emphysema may be better suited to complete the validation of the proposed genesets, unfortunately such comprehensive datasets are currently missing.

In this study, we have illustrated how text mining with an automated element can be exploited to build AOP-specific tools that can potentially be deployed for risk assessment. The text mining method used here was described by Rani et al.^16^, but is only one amongst many other possibilities. In particular, some of the filters that were used such as an impact factor cut off were arbitrary. The fluid nature of the selection criteria at the curation stage and the diversity of platforms poses a real challenge to harmonize any form of text mining strategy. The text mining exercise performed here was simple in the sense that it looked an association of genes by pairs. Yet, further information can be retrieved from the curated document corpus such as the nature of the gene pair interactions (up-regulation, down-regulation) which could give additional granularity in the downstream analyses.

In conclusion, AOPs are live constructs which evolve based on the available literature. AOPs can be used to map the most relevant end points in a risk assessment strategy. Here, we presented one approach involving manual search and automatic text mining to identify a suite of up-to-date genes associated with key events of the mucus hypersecretion AOP. Validation of the genesets conducted with in vitro samples resulted in good discrimination of a variety of treatment, IL-13, cigarette smoke, e-cigarette aerosol, and air. In vitro models combined with relevant AOP-related markers such as KE-specific genesets offer a cheaper and faster risk assessment option compared to in vivo studies. The genesets failed to predict disease status from COPD biopsy samples which is possibly confounded by technical factors and clinical factors including disease heterogeneity and medical treatment. Nevertheless, we propose that text mining is a promising and important tool that can be exploited to develop up to date AOP-related genesets.

## Materials and methods

### Stage 1: mucus hypersecretion key events literature gathering

The mucus hypersecretion AOP was described by Luettich et al*.*^12^ and the KEs were used as a basis for literature gathering. NCBI PubMed was used to access literature and followed the mining process described by Rani et al. 2015^16^. A primary body/corpus of abstracts was assembled from manual searches using the KE names (search terms can be found in Supplementary Table S1). For each KE, a list of seed genes was selected from the primary corpus by manual review. KE1 already had one seed gene defined by the AOP. Next, a document corpus was created for each KE by performing a search with each seed genes in combination with the terms “smoke OR tobacco OR acrolein OR acetaldehyde OR peroxide OR H_2_O_2_ OR reactive oxygen species” using the R package “pubmed.mineR”^16^. The document corpus for each KE is detailed in Supplementary Tables S2–S6. The HGCN compliant gene symbols present in the returned abstracts were identified by “automated named entity recognition” which is a pubmed.mineR tool to from the “term document matrix”. The abstract co-occurrence of each gene on this list with every other gene on the same list was assessed using the cosine-similarity for each gene pair^17^. Cosine similarity of gene co-occurrences was used as a score to assess potential gene interactions with 0 meaning no co-occurrence and 1 meaning 100% co-occurrence in the document corpus. Supplementary Tables S7–S11 present the cosine similarity score for each KE gene pairs at completion of stage 1.

### Stage 2: mucus hypersecretion key event literature curation

A filtering step was applied to the gene pairs occurring in each KE document corpus by retaining abstracts with HGCN gene pairs in publications with impact factor equal or above 3.0 (Supplementary Tables S12–S16). The remaining co-occurring gene pairs were automatically annotated with biological information available through four different resource databases: IntAct, KEGG, Reactome, and the Human Protein Atlas using R annotation packages^31^. The literature specified as evidence of potential biological interactions was then manually reviewed to confirm the relevance of the article. Additionally, a supervised approach was undertaken to provide further manual curation of available literature. This was conducted by reviewing the relevant reference articles cited by the authors of the manually curated abstracts, or revising the literature referenced in recent review papers. A curated document corpus was then compiled for each KE and is described in Supplementary Tables S12–S17.

### Experimental in vitro data

#### Cell culture

MucilAir™ (Epithelix Sarl, Geneva, Switzerland), a 3D cell culture systems, composed of reconstituted, differentiated human airway epithelia of nasal origin cultured the air–liquid interface were used for this study^32^. One donor was used for this study: Donor #MD046001, age: 64 yo, Caucasian, male and as the tissue was constructed from passaged cells, they were not deemed relevant material under the UK Human Tissue Act 2004. The cells were obtained with informed consent and the culture was supplied fully anonymized for research purpose only.

#### Products

1R6F reference cigarettes (University of Kentucky) are a 10 mg ISO tar yield tobacco product. 1R6F were conditioned in accordance with ISO 3402:1999^33^ before use. The prototype IS1(TT) electronic cigarette is a closed modular device and the ‘Twilight Tobacco’ variant was used containing 5 mg/ml nicotine replaceable e-liquid cartridge (Supplementary Fig. S1A).

#### Experimental design and exposure method

MucilAir™ cells were placed in exposure chambers^34^ with basal media and exposed to either aerosols from the IS1(TT) prototype or from 1R6F cigarettes. Aerosols from both products were generated using a RM20S smoking machine (Borgwaldt KC, Germany). 1R6F smoke was produced at a 1/30 aerosol:air dilution with vent blocked under the HCI smoking regime (55 ml puff volume drawn over 2 s once every 30 s with a bell-wave puff profile)^35^. Aerosol from the e-cigarette was produced at a 1/3 dilution following the CORESTA recommended method n°81 (55 ml puff volume, 3 s puff duration, 30 s puff interval), and a square wave puff profile^36^. For both exposures an air control was run alongside which received sterile air at an identical puffing regime to the product exposure. Three independent replicate exposures were undertaken for each product and air control, and within these exposures three replicate transwells were assigned per product. After exposure, cells were incubated in fresh 700 μl MucilAir™ Culture Medium for 24 and 48 hrs (RNA-seq). The basal media was collected for cell viability measurement (LDH release). The cells were lysed and stored at − 80 °C for total RNA extraction. A schematic of the experimental design is presented in Supplementary Fig. S1B.

### Experimental quality controls

The following controls were run on the samples to ensure adequate exposure to the aerosol and tissue integrity:

1. *Dosimetry* Following completion of each exposure run, media from the in vitro cell exposure chambers were collected for nicotine quantification to confirm exposure to the aerosol (Supplementary Fig. S2). The UPLC–MS/MS-based procedure is described in Haswell et al. 2017 and 2018^20,37^ with the UPLC method adapted from Onoue et al. and the quantitative MS/MS method settings described by Jin et al.^38,39^.
2. *Cellular integrity and morphology* The air control tissue morphology was checked by immunohistochemistry for the following respiratory epithelium markers: MUC5AC, FOXJ1 and p63 (Supplementary Fig. S3). To confirm health and function of the cultures at both 24 and 48 h post exposure the following markers were assessed (1) CBF (Supplementary Fig. S4), (2) trans-epithelial electrical resistance (TEER) (not shown), (3) cell viability using LDH release (Supplementary Fig. S5). All these methods were as previously described in Haswell et al*.* 2017 and 2018^20,37^.
3. *Statistics* Pairwise comparisons were assessed using a non-parametric Mann–Whitney u-test using Minitab v18.1. and non-logged data for nicotine, TEER, CBF, and LDH release.

### RNA isolation and RNA-seq

The RNA isolation and sequencing procedure was identical to the method described in Haswell et al*.* 2018^37^. Briefly, the cells were lysed with QIAzol and the RNA was extracted using the QIAGEN miRNeasy Mini Kit (Hilden, Germany) and a QIACube workstation. All RNA samples had a RIN greater or equal to 8.0. Sequencing was performed on an Illumina NextSeq 500 platform (Illumina, Sand Diego, CA, USA) at a depth of 40 million pair reads with 150 bp paired-end. (Raw FASTQ sequence files can be found on NCBI-SRA at: https://www.ncbi.nlm.nih.gov/bioproject, SRP237772).

### IL-13 and COPD treatment transcriptomics datasets

Microarray dataset GSE37693 was downloaded from the following Gene Expression Omnibus link: https://www.ncbi.nlm.nih.gov/geo/query/acc.cgi?acc=GSE37693. Briefly this geneset compares the transcriptome of the primary cell type hTECs (human Tracheal Epithelial Cells) grown at the air liquid interface and treated for 3 weeks with IL-13 and the corresponding untreated controls. The repeated IL-13 treatment triggers goblet cell hyperplasia and mucus hypersecretion^18^. The next generation sequencing dataset GSE57148 was downloaded from the sequence read archive (SRA) website: https://www.ncbi.nlm.nih.gov/sra?term=SRP041538. This dataset is derived from tissue biopsies from 98 COPD patients and 91 non-COPD smokers all diagnosed with lung cancer^19^. The biopsies were taken from the healthy margin of resected tissues.

### RNA-seq, microarray data analyses and clustering

For the RNA-seq data analysis, the assessment was performed using linear modelling with the air control exposure as reference while adjusting for relevant factors including exposure run, Next Generation Sequencing run, treatment and time point. Subsequently, empirical Bayesian analysis was applied including p-value adjustment for multiple testing, which controls for false discovery rate (pFDR)^40^. For each comparison, the null hypothesis was that there was no difference between the groups being compared. The Bioconductor package Limma was used^41^. The primary output from the statistical analysis is a set of fully annotated (when available) lists of genes differentially expressed in the comparison of interest. The same approach was applied to the COPD RNA-seq data from the Jeong et al.^41,42^ publication (PRJNA245811), but comparing COPD samples with non-COPD samples.

The normalized expression data from our 1R6F and IS1(TT) experiment and from the Alevy et al., and Jeong et al.^18,42^, papers were processed as follow for unsupervised clustering. The IL-13 microarray GSE37693 Illumina HumanHT-12 V3.0 expression beadchips probe IDs were decoded into the corresponding gene symbols using the R package illuminaHumanv3.db^43^. For all datasets the expression data was extracted for the KE genes. The data were mean-centered and scaled to unit-variance (i.e. Z-scores). The resulting samples and gene expression profiles were finally subjected to an agglomerative hierarchical clustering analysis based on their pairwise Euclidean distances and Ward linkage. Both hierarchical clustering analysis and heatmap visualization were supported by the R package ComplexHeatmap^44^.

For the COPD samples, separate PCA models were calculated for each subset of KE genes using the R package pcaMethods^45^. Pairwise association tests were subsequently performed in R between each principal component and technical factors in the data (related to read quality). For an association between a categorical and a continuous factor, ANOVA was used, whereas for an association between two continuous factors, a Spearman correlation test was used.

## Supplementary Information

Supplementary Information 1.

Supplementary Information 2.

Supplementary Information 3.

Supplementary Information 4.

Supplementary Information 5.

Supplementary Information 6.

Supplementary Information 7.
