# Supplementary material for: Application of text mining to develop AOP-based mucus hypersecretion genesets and confirmation with in vitro and clinical samples
Source: Sci Rep. 2021 Mar 17;11:6091. doi: 10.1038/s41598-021-85345-9 (PMC7969622; doi:10.1038/s41598-021-85345-9)
Supplement: Supplementary file 7 — Supplementary Information 7. [file 41598_2021_85345_MOESM7_ESM.docx]

**Application of text mining to develop AOP-based mucus hypersecretion genesets: confirmation with *in vitro* and clinical samples**

Emmanuel Minet, Linsey E. Haswell, Sarah Corke, Anisha Banerjee, Andrew Baxter, Ivan Verrastro, Francisco De Abreu e Lima, Tomasz Jaunky, Simone Santopietro, Damien Breheny and Marianna Gaca

British American Tobacco R&D Centre, Regents Park Road, Southampton, SO15 8TL UK

Corresponding author: marianna_gaca@BAT.com

**Supplementary Figure S1:** Schematic representation of the IS1(TT) e-cigarette device and cartridge in **A**. and the experimental design for aerosol exposure in **B**.

**
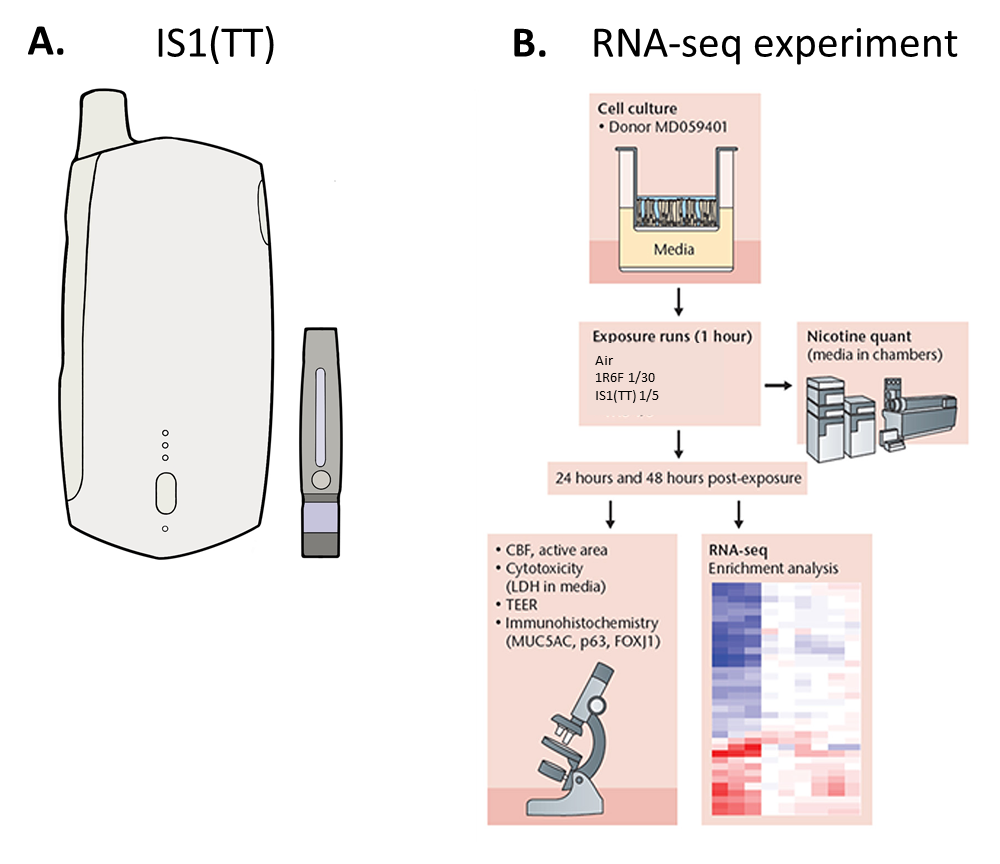
**

**Supplementary Figure S2:** Scatter dot plot of nicotine delivered during the exposure runs in the basal media of the chambers (one measurement per chamber and run). The nicotine value points for each independent experimental run (Rep 1, 2, 3) have been labelled in different colors. The chart shows the air control run with 1R6F and the air control run with IS1(TT) against nicotine in the chambers exposed to 1R6F and IS1(TT) aerosol. The mean nicotine value is shown in each chart by the horizontal line. There was no statistical difference at p<0.05 between 1R6F nicotine and IS1(TT) nicotine.


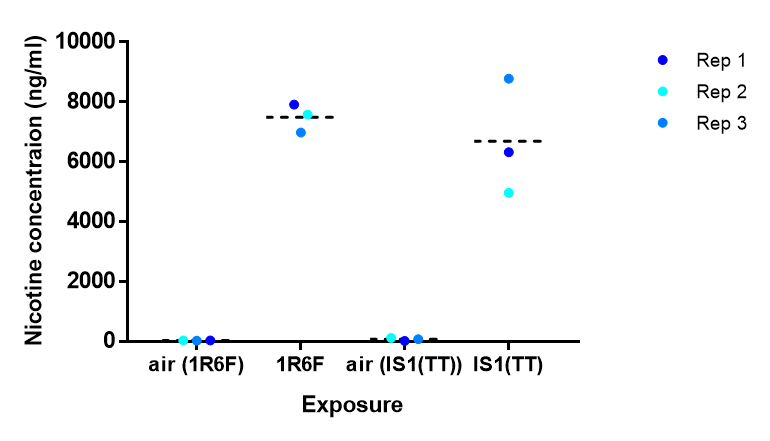


**Supplementary Figure S3:** Immunohistochemistry for FOXJ1 (marker of ciliated cells), p63 (marker of basal cells), and MUC5AC (marker of goblet cells). The cross-section was taken from MucilAir cells inserts prior exposure for quality check and to confirm the mucociliary phenotype.


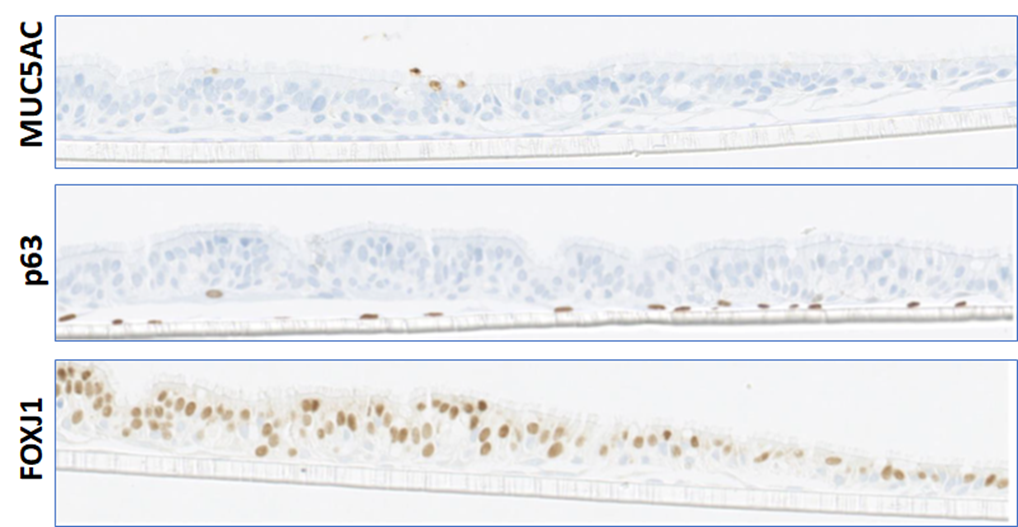


**Supplementary Figure S4**: Cilia beat frequency (CBF) single value plots. CBF single values point are shown for each independent experimental run (Rep 1, 2, 3) and they have been labelled in different colors. The chart shows the air control run with 1R6F and the air control run with IS1(TT) against nicotine in the chambers exposed to 1R6F and IS1(TT) aerosol. The CBF was measured at 24 hrs and 48 hrs post-exposure to air, 1R6F cigarette, and IS1(TT) e-cigarette aerosol. The mean CBF value is shown in each chart by the horizontal line. There was no statistical difference at p<0.05 between the aerosol treated cells and their respective air controls.

**Supplementary Figure S5**: LDH release cytotoxicity single value plots. The % of LDH release values point is shown for each independent experimental run (Rep 1, 2, 3) and they have been labelled in different colors. The chart shows the air control run with 1R6F and the air control run with IS1(TT) against nicotine in the chambers exposed to 1R6F and IS1(TT) aerosol. The LDH release was quantified 24 hrs and 48 hrs post-exposure to air, 1R6F cigarette, and IS1(TT) e-cigarette aerosol. The mean LDH release value is shown in each chart by the horizontal line. *denotes statistical difference at p<0.05.


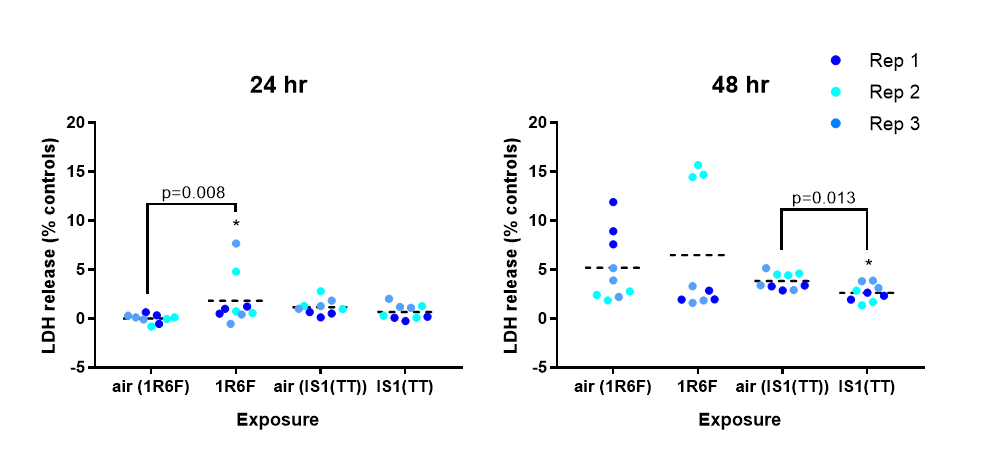


**Supplementary Figure S6:** Cosine similarity matrices for the 5 key events. The matrices represent the top 60 gene pairs based on cosine similarity score for each KE. The cosine similarity ranges from 0, genes are not found in pairs to 1, genes are always found in pairs in the document corpus. The darker the blue the higher the cosine similarity score.

**
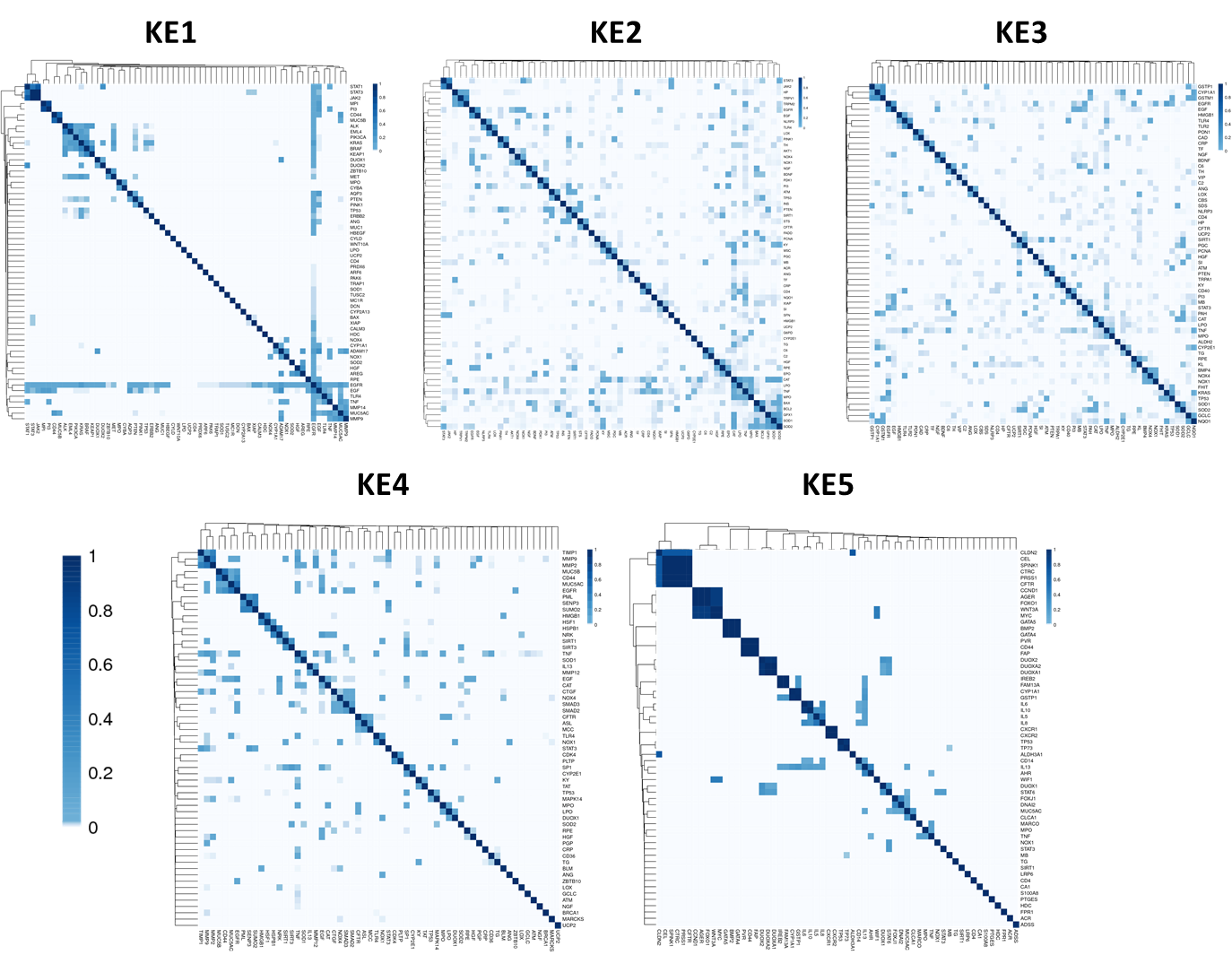
**

**Supplementary Figure S7:** Unsupervised hierarchical clustering using the KE genesets (KE1 (**A**), KE2-3 (**B**), KE4 (**C**), KE5 (**D**)) and RNA-seq data from MucilAir cells treated with 1R6F cigarette smoke, e-cigarette IS1(TT) aerosol, and air. The cells were exposed for 1hr to the aerosols and then given 24hrs and 48hrs post-exposure recovery prior RNA extraction.


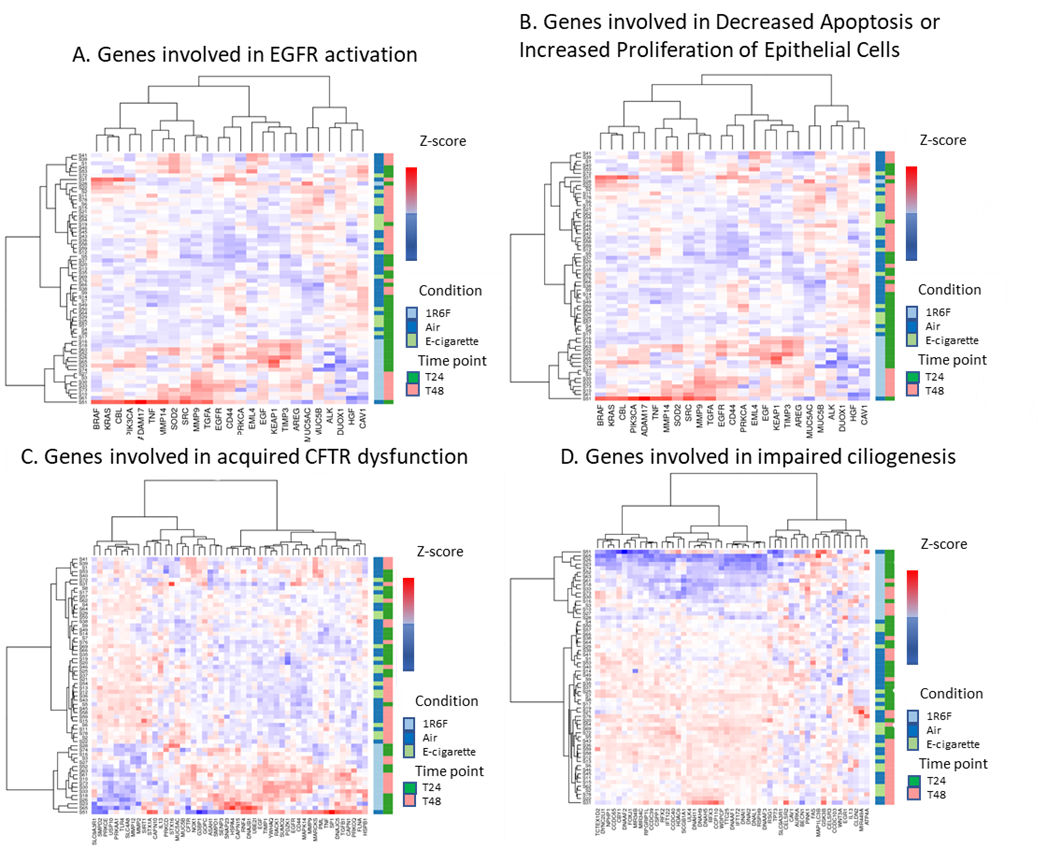


**Supplementary Figure S8:** Unsupervised hierarchical clustering using the KE genesets (KE1, KE2-3, KE4, KE5) and microarray data from human tracheal endothelial cells treated with IL-13 (IL-13) for 21 days or untreated (UT).

**Supplementary Figure S9:** Venn diagram comparing the gene overlap between a COPD geneset described by Bosse *et al.,* 2018, an asthma geneset described by Poole *et al.,* 2014, and our gene list for KE1 to 5. The table on the right lists the genes in common between these studies.
